# Haplotype association analyses in resources of mixed structure using Monte Carlo testing

**DOI:** 10.1186/1471-2105-11-592

**Published:** 2010-12-09

**Authors:** Ryan Abo, Jathine Wong, Alun Thomas, Nicola J Camp

**Affiliations:** 1 Department of Biomedical Informatics, University of Utah, Salt Lake City, UT, USA; 2 Department of Internal Medicine, University of Utah, Salt Lake City, UT, USA

## Abstract

**Background:**

Genomewide association studies have resulted in a great many genomic regions that are likely to harbor disease genes. Thorough interrogation of these specific regions is the logical next step, including regional haplotype studies to identify risk haplotypes upon which the underlying critical variants lie. Pedigrees ascertained for disease can be powerful for genetic analysis due to the cases being enriched for genetic disease. Here we present a Monte Carlo based method to perform haplotype association analysis. Our method, hapMC, allows for the analysis of full-length and sub-haplotypes, including imputation of missing data, in resources of nuclear families, general pedigrees, case-control data or mixtures thereof. Both traditional association statistics and transmission/disequilibrium statistics can be performed. The method includes a phasing algorithm that can be used in large pedigrees and optional use of pseudocontrols.

**Results:**

Our new phasing algorithm substantially outperformed the standard expectation-maximization algorithm that is ignorant of pedigree structure, and hence is preferable for resources that include pedigree structure. Through simulation we show that our Monte Carlo procedure maintains the correct type 1 error rates for all resource types. Power comparisons suggest that transmission-disequilibrium statistics are superior for performing association in resources of only nuclear families. For mixed structure resources, however, the newly implemented pseudocontrol approach appears to be the best choice. Results also indicated the value of large high-risk pedigrees for association analysis, which, in the simulations considered, were comparable in power to case-control resources of the same sample size.

**Conclusions:**

We propose hapMC as a valuable new tool to perform haplotype association analyses, particularly for resources of mixed structure. The availability of meta-association and haplotype-mining modules in our suite of Monte Carlo haplotype procedures adds further value to the approach.

## Background

Genetic studies are challenged with identifying and characterizing the underlying genetic etiology of common, complex human diseases. Recently, genomewide-association studies (GWAS) have contributed an abundance of well-replicated findings that have identified regions of the genome likely to harbor disease genes (see [1]). The current limitation is the ability to move from these initial association signals to identification of the underlying critical variants. Analytical approaches that consider haplotypes will be useful to guide the mapping of underlying variants, in particular rare variants [2,3]. Furthermore, multi-center collaborative efforts and use of resources enriched for genetic disease will be helpful in the effort to identify underlying variants. Potentially powerful family-based resources already exist for many diseases, such as those previously ascertained for linkage studies. The ability to utilize these family-based resources for haplotype association studies and combine family-based and singleton resources for joint analyses would be extremely valuable. Such analyses, however, present complex statistical challenges, such as haplotype inference and accounting for phase uncertainty in family data and the identification of appropriate statistics. Here we present a Monte-Carlo method, hapMC, designed to perform valid haplotype association analyses in mixed resources.

A significant issue for use of haplotypes in association analyses is the estimation of phase conditional on the observed genotype data. In population-based data of independent individuals, haplotype frequencies can be estimated using Bayesian methods [4]) or expectation-maximization (EM) approaches [5,6]. Such methods are well established and are scalable to thousands of markers with thousands of subjects. For family-based data, extensive work has been done to properly estimate haplotypes (see [7,8]), but there are still considerable limitations with regard to missing data and large pedigrees. Linkage analysis software, such as Genehunter [9], Merlin [10] and SIMWALK2 [11], can phase SNP data in pedigrees, however, these software either require that markers are in linkage equilibrium or cluster the markers for analysis, conditions not suited to the situation we are interested in here. Thomas (2007) [12] developed a Markov chain Monte Carlo (MCMC) linkage method that considers markers in linkage disequilibrium (LD) in general pedigrees. However, the method remains impractical for large pedigrees due to mixing problems and high computational burden. Other efforts have focused on phasing tightly-linked markers in small nuclear families and moderate sized general pedigrees [13-17]. By focusing on markers within minimum- or zero-recombinant regions these methods reduce the complexity of the haplotype reconstruction problem. Additional reductions in the haplotype configuration space are made by minimizing haplotype ambiguities and missing data with rules based on Mendelian inheritance [14,15,18,19] and genotype elimination [20,21]. Concentration on regions with minimal recombination is reasonable to address specific regions, such as candidate genes or follow-up regions identified from GWAS. However, the attention to only small to moderate pedigrees remains restrictive. Thus far, no method has integrated haplotyping strategies for SNPs in LD for larger pedigree structures with missing data.

Given any chosen method for estimating haplotypes, the uncertainty from this estimation must be accounted for at the analysis stage. For independent individuals in a classical case-control design, a likelihood approach is the usual solution, which allows consideration of all possible haplotype pairs for each individual, each weighted by the appropriate probability. For small families and transmission-disequilibrium statistics this also has been dealt with in a variety of valid ways [22-26]. Three published approaches have attempted to extend haplotype association analyses to large pedigrees and allow for combination of pedigrees and singleton data. The first approach uses a weighting scheme to account for correlation between related cases. It is limited in its requirement for independent controls when using pedigree cases and only conducts a global haplotype likelihood ratio test [17]. The second approach is an extension of a full likelihood approach for combining nuclear family and singleton data [27]. The extension to general pedigrees is by splitting these into nuclear family components and treating these components as-if independent, which can lead to invalid tests. The third is a Monte Carlo (MC) approach, proposed for single marker analyses, but with some restrictive opportunities for haplotype analyses [28]. In this MC method, to perform haplotype analyses population haplotype frequencies and phase-known observed data must be provided by the user. Haplotype inference programs can be used to provide population haplotype frequencies, because, even with related individuals, point estimates of the haplotype frequencies are unbiased for zero-recombinant regions [29]. Phase-known observed data, however, is not a realistic condition, and treating estimated haplotypes as-if phase-known is not valid [30-32]. Certainly, MC methods can be valid for association testing provided the MC procedure is performed appropriately [33,34], however, a more sophisticated approach is required than for single marker analyses.

Beyond haplotype inference and uncertainty, to perform association in pedigrees attention must be made to the controls utilized in the family data. In particular, the parents of affected offspring are intuitively not ideal for explicit use as controls because they must share exactly one allele with the affected individual. Previously, "pseudocontrols" have been suggested for family data where parents are available [22,23,35-37]. Pseudocontrols for an affected offspring can be generated from the parental alleles or haplotypes not transmitted to the affected offspring. Methods have been developed to generate up to three pseudocontrols per case to perform a matched case/control analysis and provide statistics robust to population stratification [22,23]. The use of pseudocontrols may offer more power for classical association tests in family-based resources. Data for pseudocontrols can be used in the usual association statistics, thus also providing an easy way to combine association evidence across family and case-control data - an important consideration for joint analyses in mixed resource structures.

Here we introduce an MC approach for haplotype association analyses, hapMC, which allows for valid analyses in large pedigrees and resources of mixed structure. Our method incorporates a general EM phasing method that estimates phase considering pedigree structure for a set of tightly linked markers in a non-recombinant region. Our phasing algorithm builds upon previous methods by providing a pedigree-splitting preprocessing step, a set of simplified rules optimized for SNP markers [14,15,18,19], and an integrated genotype elimination procedure in haplotype configuration construction. Valid haplotype association testing is achieved using an appropriate MC procedure, and includes full length and sub-haplotypes analyses, allowing for imputation of missing data based on the complete marker set. This new approach also allows for the use of either explicit or pseudocontrols in family data. HapMC is implemented in a Java software package, which is incorporated as a module in the freely available Genie software suite (http://www-genepi.med.utah.edu/Genie/hapMCDetail.html).

## Results

### Phasing comparison

The haplotype phasing accuracy and timing results using our pedigree-informed algorithm, the pedigree-informed algorithm HAPLORE [19] and GCHap (pedigree-naive) are shown in Table 1. Phasing accuracy was determined by the percentage of correct MLE haplotypes across all individuals. As expected, for the independent case-control data, all three algorithms produced reasonably similar accuracy results. Both pedigree-informed algorithms were marginally better (4-6% improvement) than GCHap for longer haplotypes (10 and 15 loci) due to their partition-ligation procedures; however, these marginal increases in accuracy come at the expense of increased computing time. As expected for an algorithm that is pedigree-naive, the accuracy of GCHap remained similar across all data sets, independent of the changing pedigree structures.

**Table 1:** Haplotype phasing accuracy and timing results for one data set.

			Missing data rates (%)							
			
			0		5		10		15	
			
Data	nloci	Phasing type	accuracy	time(s)	accuracy	time (s)	accuracy	time (s)	accuracy	time (s)
CC	5	new*	0.87	1.49	0.85	1.50	0.82	1.94	0.80	2.95
		
		HAPLORE‡	0.87	1.75	0.85	2.09	0.82	1.74	0.80	2.4
		
		GCHap†	0.87	1.16	0.85	1.28	0.82	1.66	0.80	1.44
	
	10	new	0.62	5.94	0.57	10.13	0.53	15.54	0.49	30.21
		
		HAPLORE	0.61	20.44	0.56	32.0	0.52	42.73	0.49	56.51
		
		GCHap	0.57	2.56	0.53	4.44	0.49	5.19	0.46	7.53
	
	15	new	0.36	42.74	0.33	122.48	0.28	316.55	0.26	1260.62
		
		HAPLORE	0.36	90.38	0.32	147.25	0.27	167.37	0.21	302.84
		
		GCHap	0.30	4.84	0.27	8.17	0.23	11.36	0.22	17.86

TRIO	5	new	0.98	1.53	0.95	1.72	0.92	2.19	0.90	2.49
		
		HAPLORE	0.98	1.47	0.95	1.50	0.92	1.17	0.90	1.47
		
		GCHap	0.88	1.15	0.85	1.38	0.82	1.54	0.80	1.60
	
	10	new	0.95	2.81	0.89	4.45	0.84	6.93	0.77	13.54
		
		HAPLORE	0.95	4.48	0.89	7.39	0.84	10.99	0.77	35.09
		
		GCHap	0.59	3.36	0.55	6.53	0.51	7.44	0.47	10.69
	
	15	new	0.92	4.52	0.81	8.15	0.73	15.13	0.65	107.28
		
		HAPLORE	0.90	12.50	0.80	56.25	-	-	-	-
		
		GCHap	0.36	7.63	0.31	11.45	0.27	16.55	0.24	29.08

ASP	5	new	0.99	1.05	0.98	1.61	0.96	1.59	0.95	2.00
		
		HAPLORE	0.99	0.61	0.98	0.74	0.96	0.60	0.95	0.67
		
		GCHap	0.89	0.98	0.86	1.35	0.84	1.40	0.81	1.43
	
	10	new	0.97	2.22	0.95	2.47	0.92	3.30	0.89	3.53
		
		HAPLORE	0.97	2.06	0.95	2.53	0.92	3.82	0.89	4.34
		
		GCHap	0.60	2.49	0.56	3.74	0.53	5.17	0.48	6.34
	
	15	new	0.93	2.99	0.91	3.64	0.85	4.5	0.80	7.90
		
		HAPLORE	0.91	3.61	0.89	32.64	-	-	-	-
		
		GCHap	0.37	5.31	0.31	7.55	0.28	9.66	0.24	15.59

LP1	5	new	0.99	2.04	0.98	1.88	0.98	1.96	0.97	2.02
		
		HAPLORE	-	-	-	-	-	-	-	-
		
		GCHap	0.87	1.60	0.86	1.64	0.85	1.69	0.85	1.82
	
	10	new	0.98	3.45	0.97	3.80	0.96	3.94	0.95	5.27
		
		HAPLORE	-	-	-	-	-	-	-	-
		
		GCHap	0.63	4.90	0.61	6.46	0.59	7.00	0.59	6.54
	
	15	new	0.96	6.76	0.95	8.23	0.93	10.85	0.92	54.92
		
		HAPLORE	-	-	-	-	-	-	-	-
		
		GCHap	0.45	8.17	0.42	9.29	0.40	10.72	0.39	15.35

*new pedigree-informed phasing algorithm

‡HAPLORE (pedigree-informed)

† GCHap (pedigree naïve)

CC (Case Control): 500 cases, 500 controls = 1000 individuals (1000 genotyped)

TRIO (both parents and one offspring): 500 trios = 1500 total individuals (1500 genotyped)

ASP (Affected Sib Pairs and parents): 250 ASPs = 1000 total individuals (1000 genotyped)

LP1 (Large Pedigree) = 5 generational pedigree ~5800 total individuals (~1500 genotyped)

- program failed.

For the data sets that included pedigree structure (TRIO, ASP, LP), the pedigree-informed algorithms achieved significantly greater accuracy than GCHap for all loci lengths and missing rates. The accuracy of both pedigree-informed algorithms continued to be similar in all situations where both algorithms completed the phasing, with our new algorithm consistently, if only marginally, the better of the two. Our new algorithm was also able to phase all data sets and scenarios generated. However, for certain scenarios with 15 loci (TRIO and ASP data sets) HAPLORE was unable to completely phase the data due to a configuration error, which was most likely due to the inappropriate removal of a critical haplotype from a partition. HAPLORE could also not be performed for the LP1 data set because it was unable to process these large pedigree data sets in a tractable amount of time. For longer haplotypes and high missing rates the improvements made by the pedigree-informed algorithms were substantial (e.g. ASP, 15 loci, 5% missing; GCHap 31% accuracy, hapMC 91% accuracy). The increased accuracy of pedigree-informed algorithms with family-based data is perhaps expected given the nature of the two approaches. Yet, the large differences in accuracies between the two types of algorithms highlights the importance of accounting for the family structure information, particularly for analyses of larger number of loci and higher rates of missing data.

Phasing time for all algorithms increased with the number of loci considered and increased missing rates, as expected. For GCHap, the phasing time increased with the number of subjects, but this increase was independent of pedigree structure. The phasing times for the CC and ASP data sets (both containing 1,000 genotyped subjects) were similar and phasing times for TRIO and LP1 data sets (both containing 1,500 genotyped subjects) were also similar. For the pedigree-informed algorithms, the number of subjects and the pedigree structure influenced the phasing time. For the CC and ASP data sets, the number of subjects is the same (1,000), however the pedigree structure in the 250 ASPs significantly reduced the haplotype configuration space, hence, phasing time is significantly reduced in the ASP data set. For example, for 10 loci, 10% missing, CC time is 15.54 and 42.73 seconds whereas ASP time is 3.30 and 3.82 seconds for our algorithm and HAPLORE, respectively. The TRIO and LP1 data sets both contained 1,500 individuals, however, the relationship between structure and phasing time is less straightforward for this comparison. The LP1 data set has more overall structure between a larger number genotyped individuals (smaller haplotype configuration space), but the pattern of the structure is more complex. Conversely the TRIO data set has less structure between total subjects (less reduction in state space), but a uniform structure across smaller units. For 0% and 5% missing data, the computing time for both TRIO and LP1 data sets were relatively similar. However, for 10% and 15% missing data (which increases the state space), the larger amount of structural information in the LP1 data set appeared to shorten the phasing time compared to the TRIO data.

Comparisons of run times between HAPLORE and our new algorithm for ASP and TRIO data sets show that HAPLORE was faster for 5 loci, but our new algorithm was faster for 10 and 15 loci, and substantially faster for many situations with 15 loci. Both pedigree-informed algorithms scaled poorly compared to GCHap when considering data sets with no or low pedigree structure (CC and TRIO), especially with larger numbers of markers and missing data. HAPLORE was markedly faster than our algorithm with 15 loci and high missing rates for CC. For example, the new algorithm was one and two orders of magnitude slower than HAPLORE and GCHap, respectively, for 15 loci and 15% missing genotype rate.

### Power and validity

Power and validity results for analyses using the simulated family and independent case-control data sets, as well as the mixed designs are shown in Tables 2 and 3. All analyses were haplotype specific tests for the known risk haplotype and were tested at the α-level of 0.05. Table 2 shows the power and type I error rates for each data set, including results from explicit controls (EC) and pseudocontrols (PC) in a standard Cochran-Armitage test for trend. In addition, the TDT was performed for the TRIO and ASP data sets. Table 3 shows the type I error rates and power for mixed resources including mixtures of two data sets.

**Table 2:** Type I error rates and power† for all data sets and statistics.

		CC*	TRIO^#^			ASP*			LP1^#^		LP2*	
		
			EC	PC	TDT	EC	PC	TDT	EC	PC	EC	PC
		
	pedigree-informed null	NA	0.046	0.044	0.045	0.052	0.051	0.051	0.049	0.047	0.054	0.054
	
	pedigree-naïve null	0.058	0.056	0.048	0.055	0.061	0.061	0.058	0.062	0.047	0.054	0.056
	
Freq risk hap	GRR											
0.17	2.0	1.000	1.000	1.000	1.000	1.000	1.000	1.000	1.000	1.000	1.000	1.000
	
	1.5	0.910	0.840	0.846	0.846	0.904	0.914	0.916	0.970	0.974	0.900	0.892
	
	1.35	0.672	0.620	0.634	0.624	0.640	0.650	0.660	0.786	0.778	0.632	0.616
	
	1.2	0.280	0.276	0.290	0.282	0.256	0.264	0.264	0.358	0.364	0.254	0.268

0.10	2.0	0.994	0.994	0.994	0.994	1.000	1.000	1.000	1.000	1.000	1.000	1.000
	
	1.5	0.754	0.712	0.722	0.726	0.728	0.732	0.738	0.896	0.900	0.762	0.742
	
	1.35	0.456	0.422	0.434	0.428	0.378	0.406	0.408	0.604	0.572	0.448	0.428
	
	1.2	0.210	0.162	0.166	0.170	0.218	0.238	0.242	0.256	0.262	0.186	0.166

0.07	2.0	0.990	0.966	0.968	0.966	0.994	0.994	0.992	1.000	1.000	0.998	0.996
	
	1.5	0.642	0.574	0.586	0.596	0.620	0.646	0.642	0.820	0.822	0.688	0.666
	
	1.35	0.399	0.300	0.312	0.312	0.368	0.400	0.396	0.554	0.538	0.368	0.360
	
	1.2	0.168	0.146	0.161	0.166	0.143	0.151	0.154	0.252	0.252	0.146	0.140

0.04	2.0	0.855	0.777	0.798	0.794	0.867	0.886	0.880	0.992	0.986	0.928	0.920
	
	1.5	0.363	0.323	0.348	0.338	0.343	0.378	0.376	0.624	0.610	0.474	0.464
	
	1.35	0.245	0.158	0.184	0.174	0.196	0.236	0.230	0.304	0.317	0.241	0.211
	
	1.2	0.096	0.139	0.152	0.141	0.087	0.114	0.104	0.152	0.128	0.118	0.112

P-values between (0.036, 0.0635) for 1000 replicates are consistent with a valid 0.05 type 1 error rate.

EC = Explicit controls, PC = pseudocontrols, TDT = transmission disequilibrium test

* = 500 cases, 500 controls

# = 500 cases, 1,000 controls

† Power is shown for the hapMC with the pedigree-informed MLE estimation

NA = pedigree informed phasing not applicable to case-control.

**Table 3:** Type I error rates and power† for mixed resource study designs.

		CC	TRIO	ASP	LP1	LP2				
						
		EC	PC	PC	EC	EC	TRIOCC	ASPCC	LP1CC	LP2CC
		
	Total sample size	1,000*	1,500†	1,000	1,500	1,000	2,500	2,000	2,500	2,000
	
	pedigree-informed null	NA	0.044	0.051	0.049	0.054	0.051	0.039	0.042	0.044
	
	pedigree-naive null	0.058	0.048	0.061	0.062	0.054	0.053	0.046	0.048	0.060
	
Freq risk hap	GRR									
0.17	2.0	1.000	1.000	1.000	1.000	1.000	1.000	1.000	1.000	1.000
	
	1.5	0.910	0.846	0.914	0.970	0.900	0.996	0.998	0.996	0.998
	
	1.35	0.672	0.634	0.650	0.786	0.632	0.904	0.912	0.934	0.918

	1.2	0.280	0.290	0.264	0.358	0.254	0.516	0.518	0.566	0.500
	
0.10	2.0	0.994	0.994	1.000	1.000	1.000	1.000	1.000	1.000	1.000
	
	1.5	0.754	0.722	0.732	0.896	0.762	0.950	0.960	0.984	0.978
	
	1.35	0.456	0.434	0.406	0.604	0.448	0.744	0.730	0.804	0.788
	
	1.2	0.210	0.166	0.238	0.256	0.186	0.324	0.390	0.372	0.358

0.07	2.0	0.990	0.968	0.994	1.000	0.998	1.000	1.000	1.000	1.000
	
	1.5	0.642	0.586	0.646	0.820	0.688	0.866	0.904	0.952	0.944
	
	1.35	0.399	0.312	0.400	0.554	0.368	0.598	0.674	0.752	0.678
	
	1.2	0.168	0.161	0.151	0.252	0.146	0.250	0.264	0.336	0.294

0.04	2.0	0.855	0.798	0.886	0.992	0.928	0.981	0.990	0.998	0.998
	
	1.5	0.363	0.348	0.378	0.624	0.474	0.618	0.616	0.746	0.726
	
	1.35	0.245	0.184	0.236	0.304	0.241	0.340	0.411	0.430	0.429
	
	1.2	0.096	0.152	0.114	0.152	0.118	0.240	0.167	0.200	0.176

Results from most powerful statistic for each single resource analyses and mixed resource analyses. All controls within LP1 and LP2 are familial controls.

* = 500 cases, 500 controls

† = 500 cases, 1,000 controls

† Power is shown for the hapMC with the pedigree-informed MLE estimation

NA = pedigree informed phasing not applicable to case-control null.

Based on 1,000 replicates, all type I error rates were found to be not significantly different than 0.05 (95% confidence interval [0.036, 0.064]), indicating validity of all tests within the MC framework both for hapMC using the pedigree-naïve and pedigree-informed MLEs. Primarily, these results demonstrate the versatility and potential for hapMC to perform valid analyses on mixed structure study designs.

For the TRIO and ASP data sets, we performed trend tests using EC and PC designs and also a TDT analysis. In general, the ASP data set exhibited more power than the TRIO data set even though it had a smaller overall sample size (1,000 versus 1,500), presumably due to the enrichment of disease alleles in the ASP set. The exception was at the low genotypic relative risk of 1.2, where the increased sample size of the TRIO design appears to have out-weighed the minor genetic enrichment of the ASPs at this small risk size. Within both data sets power was observed to be quite similar across the three analysis approaches for all alternative models. However, both PC and TDT statistics showed consistently higher power than the EC, although these gains were extremely marginal (≤3.5% increase in power). Formal testing of the differences between the PC and EC approaches using a Wilcoxon signed-rank (WSR) test provided evidence for significant differences (TRIO: PC v EC p_WSR _= 0.001; ASP: PC v EC p_WSR _= 0.0012) indicating consistent marginal power gains when using PC compared to EC in these designs. The power from the PC and TDT statistics differed by no more than 1% in the TRIO data set and by less than 2.2% in the ASP data set and were not statistically different (TRIO: TDT v PC p_WSR _= 0.392, ASP: TDT v PC p_WSR _= 0.168).

For the LP data sets we also compared the EC and PC approaches. In contrast to the TRIO and ASP data sets, the PC approach in large pedigrees involves a mixture of pseudo and explicit controls according to the pedigree structure. If both parents of an affected case are genotyped and are unaffected, then a pseudocontrol is generated from their data and used in place of their explicit data, otherwise controls are considered explicitly. In the LP1 data set, power differences between the EC and PC approaches were marginal across all models and neither approach was consistently better than the other. The largest difference between the two was 3.2% for the disease model with a risk haplotype of 0.10 and a GRR of 1.35. Formal testing indicated that there was no evidence that one approach was consistently superior to the other (p_WSR _= 0.28). However, in the LP2 data set, the EC approach most often gave marginally more power (in all but one model) with an average increase of 1.35%. This consistent marginal increase was significant (p_WSR _= 0.006).

Examining power across all resource designs shows that the LP1 data set, matched in sample size to the TRIO data set, was consistently more powerful than the TRIO data set for all risk haplotype frequencies and GRRs (p_WSR _= 0.009). The maximum difference in power of 26.2% between the two data sets is seen with the 0.04 risk haplotype frequency with 1.5 GRR. Furthermore, even though the other three designs (CC, ASP and LP2) had smaller sample sizes, these designs also out-performed the TDT design for the majority (at least 75%) of models. The LP2, matched in sample size to the CC and ASP data sets, performed comparably to the CC or ASP data sets (both p_WSR _>0.3). An increase in power of the LP1 compared to LP2 was evident (p_WSR _= 0.001) consistent with the increase sample size of LP1 which contains twice as many controls.

For the mixed nuclear family and case-control designs that include TRIOs and ASPs (TRIOCC and ASPCC), the superiority of the PC approach reflected the observations for the single data set results (TRIOCC: PC v EC p_WSR _= 0.008, ASPCC: PC v EC p_WSR _= 0.0075). In the mixed large pedigree and case-control data sets, the PC approach outperformed the EC approach in one set (LP1CC: PC v EC p = 0.036), but not in the other (LP2CC: EC v PC p = 0.61). Given the superiority of PC to EC in all but LP2CC and the marginal nature of the individual differences, only the PC results are detailed in Table 3.

As expected, power was always increased in the joint, two-data-set mixed resources compared to either single data set. Furthermore, and previously shown by others [38], the power of the joint analysis in the mixed resource was always superior to the power of analyzing both resources separately (power = 1-{(1-p_1_)×(1-p_2_)}, where p_1 _is the power for the first data set and p_2 _is the power of the second).

## Discussion

Here we have described a MC, MLE-based haplotype association method and software (hapMC) designed to analyze a set of tightly-linked SNPs in general pedigree and/or independent case-control based studies. HapMC allows for the haplotype MLE to be estimated by either a pedigree-naïve or pedigree-informed algorithm. A novel aspect of our method comes from the implementation of the pedigree-informed general phasing algorithm that appropriately handles related and unrelated individuals into the haplotype phasing. A variety of pedigree-informed phasing algorithms currently exist (see [7,8]), but none have established practical measures for dealing with large amounts of missing data in extended pedigrees and directly integrated these for haplotype association testing. Our algorithm includes a preprocessing step to optimally split large pedigrees into substructures, which enables it to consider important pedigree structure that surrounds dense genotype data and maintain tractability. While this step may appear trivial, it is a necessary step to analyze large pedigrees with missing data that current phasing programs cannot handle. Our new approach includes both incorporation of pedigree structure and a partition-ligation in the haplotype estimation procedure.

We found that the accuracy of haplotypes estimated from our pedigree-informed algorithm was always equal or superior to that estimated without these algorithm improvements. Even in the situations where there was little or no pedigree structure (CC and TRIO data sets), the new algorithm performs substantially better due to the partition-ligation alone. However, a notable issue from our investigations is that while the new pedigree-informed algorithm always results in greater accuracy than a pedigree-naïve approach, the phasing times are orders of magnitude longer for data sets with little or no pedigree structure, large number of loci (nloci = 15) and high missing rates (10-15%). Hence, for incorporation in a Monte Carlo analysis approach where the procedure must be repeated thousands of times, the new phasing algorithm is impractical for unrelated individuals with high missing rates. We therefore recommend that for a resource of unrelated individuals (CC data sets) the standard full likelihood approach (as implemented, for example, in [27]) is the best alternative. However, for data sets that include family structure we find that substantial haplotype accuracy is lost by ignoring pedigree structure, and the use of an algorithm that considers that structure (such as hapMC) is a more prudent choice. In some situations, this may require a more stringent quality control protocol with higher minimum genotyping thresholds to retain practical application of the more sophisticated algorithm.

Comparisons between our new phasing algorithm and a previously proposed pedigree-based algorithm, HAPLORE [19], show equivalent or slightly better MLE haplotype accuracy for the new algorithm for all situations and data sets considered. In terms of phasing times, our algorithm ran substantially faster in most situations considered for comparison, particularly when using family-based data with higher number of markers and missing rates. Furthermore, HAPLORE was unable to be used for in nuclear families with larger numbers of loci and high missing rates, or in the large pedigrees.

As an empirical approach, the space and time requirements for hapMC can be considered limitations. The time required to phase haplotypes and calculate the observed association statistics must be scalable to be able to practically generate the necessary MC simulations. As has been mentioned, the haplotype phasing aspect of hapMC can be computationally intense for large data sets and high missing rates. For example, to analyze ten markers in one mixed large pedigree and case control resource with ~1500 genotyped individuals the hapMC algorithm required ~21 minutes using a 2.40 Ghz processor and 2 Gb of memory. These requirements increase with increased missing data and markers and decreased pedigree structure. In our current application, the total number of loci hapMC can practically handle is approximately 20, although the precise limitation is dependent upon the data set characteristics (including missing data rate, number of individuals and families, and types of families). However, because the method is designed for tSNPs across a non-recombinant follow-up GWAS region or candidate gene, marker sets of fewer than 20 markers is not unreasonably small. The marker limitation is also consistent with other programs with similar approaches.

It is known that the use of MLE haplotypes in association analyses (that is, ignoring phase uncertainly) can lead to invalid association tests and may result in biased estimates of effect size and other parameters [30-32]. We emphasize here that all tests in hapMC are under the null hypothesis of no association of any haplotype, and that the key to the MC procedure in producing valid association statistics using the MLE haplotypes is to generate properly matched null data from which to generate the null distribution [34]. Our method uses a MC procedure that matches the entire phasing process and the use of MLEs in the observed data and in all null data sets used for the null distribution. HapMC therefore produces accurate significance levels for both tests for independence and effect size, as we have shown. However, the point estimates for effect size statistics (that is, odds ratios) estimated from our method may be upwardly biased. Such biased effect sizes are possible when using pedigree data that have been ascertained for disease and analyzing related controls explicitly. While the bias may be removed by using a matched case/pseudocontrol analysis within families [39], the point estimates (such as odds ratios) should be interpreted with caution both due to the use of MLE and the pedigree-based data. It is worth noting that in joint analyses of multiple resources, if the disease MAF and/or disease effect size is anticipated to differ across the component studies that a formal meta procedure should be followed. HapMC has been incorporated in to the Genie framework, and hence formal meta procedures can be implemented in the approach [40].

Not addressed here, is that while haplotype association testing is considered a reasonable approach to explore, it is often burdened with the task of determining which haplotypes or sub-haplotypes that should be tested. It may be of interest to note, that the MC, MLE haplotype association approach outlined in this paper has also been incorporated into a peripheral Genie software package called hapConstructor [41] (http://bioinformatics.med.utah.edu/Genie/hapConstructor.html). HapConstructor is a data mining software aimed at identifying the most significant haplotypes from a data set. However, due to computing time constraints of a data-mining approach, currently hapConstructor is limited to using the phase-naïve EM algorithm for haplotype estimation. Mixed resource structures and formal meta analyses are supported within hapConstructor and, as we have shown here, even though it may not be ideal, our MC approach with the pedigree-naïve MLEs remains valid.

We have illustrated hapMC using multiple single data sets of varying design, as well as several joint resources based on a combination of one traditional case-control data set and one family-based data set. However, the MC approach extends more generally to multiple constituent groups where each can be from any study design. Furthermore, the family structures it can analyze are not limited in size or structure. This feature was demonstrated here by the LP data sets that were five-generation pedigrees with substantial missing data. We re-emphasize that large pedigrees with missing data may necessitate pedigree splitting at the phasing step, but that the full structures are maintained when generating the null configurations to fully account for the familial relatedness in the association analyses. To our knowledge, hapMC is the only method and software currently available that can provide valid haplotype analyses in resources of mixed study designs that include general pedigrees. As previously shown by others [38], the importance of joint analyses is the increased power such analyses offer over simply combining the statistical evidence from two separate analyses.

Beyond demonstrating the validity of the method, our power results provide some insight into the relative strengths of different study designs and statistical approaches. In our single data set analyses, for TRIO and ASP data sets we found that both the trend test with a PC approach and the TDT were superior to the EC approach. There was a lack of significant difference in power between the PC approach and the TDT analysis. From this we would conclude that the TDT statistic remains the preferred statistic for analyzing nuclear family designs due to its additional robustness to population stratification. In the large pedigree data sets, the relative superiority of the two approaches (PC and EC) was not clear. In the single data sets (LP1 and LP2), the EC and PC approaches were similar in LP1 and the EC approach appeared superior in LP2. In the joint data sets (LP1CC and LP2CC), the PC approach was superior in LP1CC, but no significant difference was found in LP2CC. The lack of impact of the PC approach in LP2 may be due to the reduced control size in that data set (1,000 vs 1,500 total), however, our observations highlight the difficulty in defining optimal approaches for general pedigrees where the specific structure may influence the relative powers of different approaches. To investigate this we repeated our LP analyses, but with oversampling of parents of affected cases and less sampling of other individual as controls, thus increasing the number of occurrences that the pseudocontrol could be used in the analysis. We found that the PC approach improved in power (data not shown). In summary, the PC approach was found to have significant superiority over the EC approach in the TRIO, ASP, TRIOCC, ASPCC, and LP1CC data sets. Only one design indicated superiority of the EC approach (LP2), and the remainder indicated no significant difference (LP1, LP2CC). Our results therefore suggest that the PC approach is likely to be the better approach for mixed nuclear family and case-control designs.

We also explored a stricter definition to select familial controls. We found that if close relatives are simply not considered in the analyses (restrict controls to only those further than first degree) that the power was adversely affected by the reduced control sample size (data not shown). This indicates that the close relatives contribute positively to the power of the analysis.

Comparing different single study designs, the TRIO design consistently performed worse than all other designs. Of the remaining designs, it was interesting to note that for matched sample sizes the large high-risk pedigree design (LP2) was comparable in power to ASP and CC designs. Large pedigrees arguably contain the most redundancy in the familial cases (and controls), but are also enriched for disease alleles (ascertainment criteria of 14 cases required per large pedigree, a rate of 2.0 fold increase over the sporadic rate). Familial controls have previously been shown to increase power [42,43]. The comparability in power between LP2 and CC suggests that the positive effect of the disease allele enrichment in LP2 may have balanced the decrease in effective sample size due to the redundancy in information from related subjects. We also found that LP2 and ASP were not significantly different for power and both designs are enriched for disease. However, on average, large pedigree controls are less related to the cases than controls are in ASPs, hence the effective increase in control population in LP2 may balance the effect that the effective sample size of the cases is reduced. Of course, it must be noted that our results may be specific to our simulated data sets, and, for other large pedigree structures, these findings may not hold. Nonetheless, our results indicate substantial potential for large pedigree resources and using pedigree-based controls for haplotype association analyses.

## Conclusions

In conclusion, we have developed a method and software to perform valid haplotype analyses in resources of mixed pedigree structure. To our knowledge this is the only method currently available that can perform such analyses. Similarly to that found by others [38], our findings illustrate the power advantage of joint analyses and, furthermore, suggest family-based resources can play a valuable role in haplotype association studies.

## Methods

### Haplotype phasing

We have implemented a general haplotype phasing algorithm designed to estimate population haplotype frequencies as well as determine maximum likelihood estimate (MLE) haplotype pairs for a set of tightly linked markers in general pedigrees. It can also be used for singleton data (independent cases and/or controls). The method involves three parts: (1) data preprocessing; (2) identification of all possible haplotype configurations in pedigrees; (3) and an EM algorithm across the haplotype configuration state space to estimate haplotype frequencies and MLE haplotype pairs.

#### Part 1: data preprocessing

Although the algorithm is general to pedigree structure, the missing data inherent in large pedigrees may make it intractable to consider all haplotype configurations for the total structure. To address this we have developed a preprocessing algorithm to determine the sub-structures within large pedigrees to retain for phasing. Our algorithm selects these substructures to maintain tractability. The algorithm works by determining all the nuclear families within the full pedigree. For each nuclear family, if both parents and at least one offspring have sufficient genotype data (a user-defined parameter) then the nuclear family unit is maintained for the phasing process. After iterating through all nuclear families, those selected for retention are connected back together if overlapping individuals exist. Individuals that are part of non-retained nuclear families are considered as independent individuals, or alternatively are removed from the analysis if they do not have sufficient genotype data. This process is designed to remove pedigree structure that will lead to a prohibitively large haplotype configuration space for the EM algorithm, while also maintaining as much pedigree structure as possible. Only the substructures identified are integrated into the estimation of the MLE haplotypes and haplotype frequencies. However the full pedigree structure is always maintained for the statistical analysis in the MC procedure, such that the correction for all known relationships is maintained in the analyses.

Also involved in the data preprocessing is an iterative process whereby a series of rules are used to reduce phase ambiguities and missing data across all markers within each pedigree. We assume a zero-recombinant autosomal region and mutation- and error-free SNP data. All parent-offspring trios that were maintained for phasing are considered with these rules, which are repeated iteratively until no more updates can occur. The four steps involved in this part of the preprocessing are detailed below. In step 1, genotypes are loaded into variables efficient for updating. Steps 2 and 3 are designed to use known homozygous genotypes to resolve both parent and child unphased and missing locus positions. Step 4 considers the parent-offspring trios to further reduce phase ambiguities based on basic rules of inheritance and transmission. Step 1 is performed once per individual, steps 2 and 3 are performed once per parent-offspring trio, and step 4 is repeated until no further updates can be completed.

##### Step 1 - Load haplotype variables

This step reads each individual's genotype data into six variables that are used to fully define and store the genotype data. Each of these variables is an *n*-length string of '0' and '1' values, or bits, with the *i*^th ^position in the string storing information for the *i*^th ^SNP locus. The value '1' indicates that a condition is satisfied, '0' that it is not. The first two variables indicate the 'heterozygous' and 'homozygous' status of each locus. The third is the 'unphased' variable that indicates whether the data at locus *i *remains unphased (1) or has been phased (0). These three variables apply to a haplotype pair and thus there is only one of each of these defined per individual. The remaining three variables are haplotype specific and hence two of each are defined per individual -one for each haplotype. The fourth 'set' variable indicates whether the allele at locus *i *has been assigned. The fifth 'missing' variable indicates whether the allele at locus *i *is missing. The sixth 'value' variable indicates whether the allele at locus *i *is the minor allele.

Storing genotype data in this way allows for comparisons between individuals and updates to be performed quickly using bitwise operations that can consider the full set of loci simultaneously, rather than iterating through each of the *n *loci separately, and is therefore computationally efficient. Table 4 shows an example of how genotype data are loaded into these variables.

**Table 4:** Example of preprocessing step 1, loading genotype data into the six n-locus bit variables (n = 5).

	M1	M2	M3	M4	M5
Genotype values*	12	00	11	12	22

**Variables**					

**Haplotype pair**					

Homozygous†	0	0	1	0	1

Heterozygous§	1	0	0	1	0

Unphased**	1	0	0	1	0

**Haplotype 1**					

Set‡	0	0	1	0	1

Missing††	0	1	0	0	0

Value§§	0	0	0	0	1

**Haplotype 2**					

Set	0	0	1	0	1

Missing	0	1	0	0	0

Value	0	0	0	0	1

*11 indicates a homozygous genotype for the major allele; 12 a heterozygous genotype, and 22 a homozygous genotype for the minor allele; 00 indicates missing genotype.

†Homozygous 0 indicates the positions that are heterozygous or missing and 1 indicates the homozygous positions.

§Heterozygous 0 indicates the positions that are homozygous or missing and 1 indicates the heterozygous positions.

**Unphased 0 indicates the positions that are phased or missing and 1 indicates a heterozygous position without known phase.

‡Set 0 indicates the positions that have not been assigned an allele (i.e. unphased or missing) and 1 indicates the allele value and phase is known.

††Missing 0 indicates the positions that have an observed allele value and 1 indicates positions that are missing.

§§Value 0 indicates the positions that have the major allele or unknown and 1 indicates positions that have the minor allele.

The six variables hold all the pertinent pieces of information for phasing. Clearly, if the variables 'missing', 'unphased', and 'set' are 000..0, 000..0 and 111..1, respectively, then the haplotype has been completely specified at all loci and is unambiguously determined. In step1 we initialize the variables simply based on the individuals own data, and then in steps 2-4 we use any parent-offspring relationships to remove as much ambiguity as possible. That is, the variables are updated towards the fully unambiguous state. Of course, many positions will remain ambiguous after this process. These become the positions that are iterated through to identify all possible haplotype configurations in Part 2, which subsequently defines the state space for the EM algorithm in Part 3. To help the updating process we additionally define inheritance and transmission single-bit variables. These variables indicate which haplotypes are shared by the parent-offspring pair in the current variable states. This provides the basis for determining which haplotypes to transfer information between during updates. For each parent-offspring pair, one transmission and two inheritance (one for each offspring haplotype) variables are defined. The transmission variable indicates which parental haplotype is shared with the offspring (value = 0 if haplotype 1 is transmitted and shared, 1 if haplotype 2 is transmitted). An inheritance variable indicates whether an offspring haplotype is shared with the father or the mother (value = 0 if haplotype is inherited from and shared with father, 1 if inherited from mother). Along with the six genotype variables, the transmission and inheritance variables are updated and reassessed as the haplotype states change in steps 2-4.

##### Step 2 - Parent-to-offspring homozygous updates

With this rule, parental homozygous loci are used to resolve phase ambiguities in the offspring's haplotype. If an offspring has not yet been updated from a prior parent-offspring update (either as a parent or an offspring), then either of the offspring's haplotypes can be chosen to be updated. If the offspring was previously updated in the parent-to-offspring pair involving the other parent, then the offspring's inheritance variables will be assigned. If the offspring was previously updated as a parent, its inheritance variable will not be assigned but the haplotype configurations will be uniquely defined. In this instance, the inheritance rule is applied (see Table 5) to determine which haplotype to update. If the inheritance rule is inconclusive no update is made.

**Table 5:** Inheritance and transmission rules.

Rule	Description
Inheritance	Indicates which haplotype is received by the offspring (characteristic of offspring haplotypes).
	
	The parental source of an offspring haplotype can be established using exclusion. That is, once an offspring haplotype is known not to be from one parent, it is must be from the other parent.
	
	Exclusion can be determined if a haplotype has an allele not found within a parent's genotypes or the haplotype does not match either of a parent's set haplotypes.

Transmission	Indicates which haplotype is transmitted by the parent (characteristic of parental haplotypes).
	
	Which haplotype is transmitted from a parent to an offspring can be established using exclusion. That is, once a parental haplotype is excluded as being either haplotype in an offspring, then the alternate parental haplotype must be the transmitted one.
	
	A conditional exclusion can be determined by examining the situation where one parental haplotype was transmitted to the offspring and check if the complimentary haplotype from the offspring's genotypes could be inherited from the other parent.

**Figure 1:**
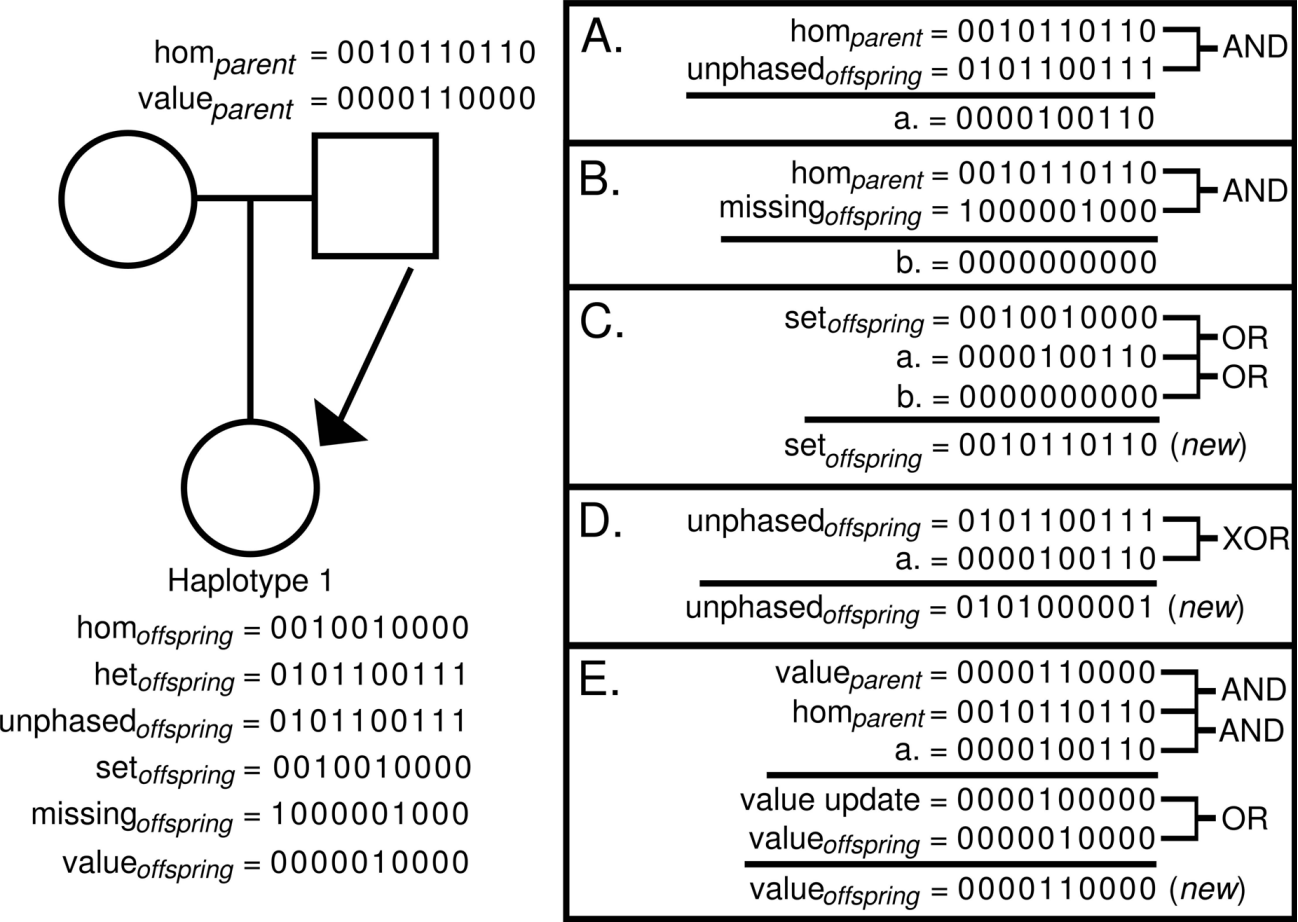
**Example of a parent-to-offspring homozygous update using bit-variables**. The parent's homozygous variable (hom_parent_) is used to update variables in the offspring. In this example, offspring haplotype 1 has been chosen for update. Variables listed on the left in the trio drawing are the current states for the offspring and parent. Variables listed in the panel on the right and indicated by (new) are the updated states. Panel A. Logical "AND" operation determines which loci are homozygous in the parent and unphased in the offspring. The result (a.) indicates positions (value = 1) where updates can be made to the set_offspring_, value_offspring _and unphased_offspring _variables. In this example, 3 positions can be updated (the 5^th^, 8^th ^and 9^th^). Panel B. Similar to panel A, but for the missing_offspring _variable. In this example, no positions can be updated for this variable (all position in b. = 0). Panels C-E. Logical operations "OR", exclusive OR ("XOR") and "AND" are used to determine the new updated versions of variables set_offspring_, unphased_offspring _and value_offspring_.

The update involves establishing if any loci in the chosen offspring haplotype are missing or unphased where the parent is homozygous. If so, these positions are updated in the offspring haplotype variables (set, unphased, missing, and value variables) using logical bitwise operations. Figure 1 illustrates the logical bitwise operations that take place in a parent-to-offspring homozygous update. Once the update is made to the variables for the chosen haplotype, the inheritance variables for both offspring haplotypes (for the specific parent-offspring pair) are assigned.

##### Step 3 - Offspring-to-parent homozygous updates

With this rule, the offspring homozygous variable is used to resolve ambiguities in the parent's haplotypes. Similarly to step 2, for each parent-offspring pair, a parental haplotype is selected for update. This is determined by checking if the parent has been updated from a prior offspring-to-parent update (either as a parent or offspring). If the parent has not been updated yet, either haplotype is chosen to be updated. If the parent has been considered as an offspring in an offspring-to-parent pair, then the transmission will not be assigned but the haplotype configurations will be uniquely defined. In this instance, the transmission rule is applied (see Table 5) to determine which haplotype to update. If the transmission rule is inconclusive no update is made.

The update involves establishing if any loci in the chosen parent haplotype are missing or unphased where the offspring is homozygous. If so, these positions are updated in the parent haplotype variables (set, unphased, missing, value variables) using logical operations. Once the update is made to the parent haplotype, the transmission variables for this parent to the specific offspring are assigned.

##### Step 4 - Reduce haplotype phase ambiguities

For further reducing haplotype phase ambiguities we have implemented and built upon the concepts outlined in rules 3-13 in Zhang et al. [19]. These rules consider parent-offspring trios and work by iteratively updating the inheritance and transmission states between the offspring and each parent, which allows ambiguity reduction between offspring and parent haplotypes. The procedure starts by first attempting to resolve the unknown transmissions from both parents to an offspring, and then resolving unknown offspring inheritance states. In our implementation, we have reordered rules 3 and 4, and 5 and 6 from Zhang et al. [19] so that known transmissions from both parents can be used to help determine inheritance. That is, if one parents' transmitted haplotype is known and one of the offspring haplotypes is known to not be equal to it, then transmission can be established and the offspring inheritance variables can be assigned. The remaining rules (7-13) are implemented as previously described [19]. Rule 7 is applied when the shared haplotype between a parent and offspring is known and either copy of this haplotype can be used to update any ambiguities in the other. Rules 8 and 9 are applied when the either the inheritance or transmission for a parent and offspring is known but not both. When neither inheritance nor transmission is known, then rules 10 and 11 are applied. Lastly, rule 12 is used to reset homozygous positions that were altered from the previous rules, and rule 13 sets the phase of one heterozygous locus if all other set alleles are homozygous. As for steps 2 and 3, all rules are implemented as logical operations.

#### Part 2: Identification of all possible haplotype configurations

After Part 1 is complete, the variables may still contain unknown or unphased positions. Expansion to all possible values for these positions will generate all possible haplotype pairs for an individual. As the possible haplotypes are enumerated for each offspring in a nuclear family, the nuclear family configurations are established. Rather than creating a separate step for the haplotype elimination process [20,21], we have integrated this directly into our procedure for creating the configurations. This is done by iterating through each offspring in the nuclear family. For the first offspring, all the haplotype possibilities for this offspring that are compatible with the parent haplotypes are used to create a nuclear family haplotype configuration. For the remaining offspring, we iterate through the haplotype possibilities and add compatible configurations or discard incompatible configurations. We perform this starting from the offspring with the minimum number of haplotype possibilities that allows us to limit the number of possible configurations created and stored. After creating all nuclear family configurations, the full haplotype configurations are assembled for the pedigree substructures chosen in our preprocessing step (that is, structures containing multiple connected nuclear families). This step works by matching together all the nuclear family configurations through the linking individuals.

#### Part 3: EM algorithm for haplotype frequency and MLE haplotype estimation

An EM algorithm is used to maximize the likelihood of the haplotype frequencies given the observed genotype data and pedigree structure under the assumption of Hardy-Weinberg equilibrium (HWE). Consider a pedigree with *m *members with marker phenotypes ***y ***and population haplotype frequencies ***H. ***Among the *m *members there are *f *founder individuals and *d *descendants, *m *= *f *+ *d*. Each individual has a set of haplotype pairs consistent with their marker phenotype data, *y*_i_, which resolve to multiple pedigree haplotype configurations, **c**, as determined in Part 2. Each possible configuration contains a set of haplotype pairs (h_*1*_, h_2_, ... , h_*m*_) across each individual in the pedigree. The haplotype pairs consist of a maternal and paternal haplotypes, h_*i *_= (h_*mi*_, h_*pi*_). The likelihood for each pedigree is defined from the Elston-Stewart algorithm [44] is:

$$L\left(y|H\right)=\sum_{c}\prod_{f}P\left(h_{f}|H\right)\prod_{d}P\left(h_{d}|h_{mi},h_{pi}\right)$$

For the founders, the probability of the haplotype pair, *P*(*h*_*f *_*|H*), is calculated according to HWE as the product of the corresponding haplotype frequencies if the haplotypes are equal or double the product if they are unequal. For descendants, the gametic transmission probabilities, *P*(*h*_*d *_| *h*_*mi*_, *h*_*pi*_), are calculated based on Mendel's laws. The overall likelihood is the product across all pedigree likelihoods. The EM is an iterative process that alternates between an expectation or E-step and a maximization or M-step. The E-step estimates the probability of the haplotype configurations given the current haplotype frequency estimates. Based on the haplotype configuration estimates, the expected count for each haplotype is derived. This is done by counting the occurrences of a haplotype in a configuration and weighting this count by the probability of the haplotype configuration in the family (or individual) of which it occurs. The M-step updates the haplotype frequency estimates based on the expected haplotype counts. The iterations continue until the difference in the estimates between iterations is less than a user-defined value.

To further reduce the complexity and state space for possible haplotype configurations, we have also implemented a partition-ligation strategy in conjunction with the EM algorithm (PL-EM) [45]. The PL-EM technique works by splitting the complete marker set into smaller overlapping marker sets with a user-defined number of markers. For these smaller partition lengths, the haplotype configurations are assembled and the haplotype frequencies are estimated using the EM algorithm. When two adjacent units have been completed, they are ligated and the procedure is re-applied. The size of the haplotype configuration state space is reduced by removing haplotypes with frequencies below a set threshold within each partition. The reduction in haplotypes in each partition limits the subsequent set of possible haplotype configurations in the ligation step.

### Association Testing

HapMC has been developed to allow the user to test specific hypotheses of individual SNPs, sub-haplotypes (any subset of the full SNP set) and full-length haplotypes. The hapMC module is integrated into the Genie software package [28] allowing the use of all the test statistics provided by Genie for dichotomous and quantitative outcomes. For dichotomous outcomes, these are the classical association test statistics for risk and non-independence (odds ratio, chi-squared, and chi-squared trend) which can be tested based on haploid or diploid data. Haploid models are allele-based (or haplotype-based) tests where the unit of interest is the chromosome. Diploid models are genotype (or paired-haplotype) tests where the unit of interest is the individual. Also, the TDT, sibling-TDT and combined-TDT transmission-disequilibrium test statistics are available. Here, we have added the option to generate pseudocontrols for genotyped cases where both parents of the case are genotyped [35-37]. For haplotype tests, the MLE haplotype pair can be estimated by ignoring all familial relationships or using the phasing algorithm described above. The haplotype pair for a pseudocontrol is composed of the two parental haplotypes not transmitted to the genotyped case. Haplotypes for pseudocontrols are then used in the standard way with the aforementioned dichotomous case-control statistics. For quantitative outcomes, the quantitative TDT, analysis of variance, and differences in means test are available. If multiple populations are present, or a difference of effect size is suspected across the multiple data sets considered, options are available to estimate haplotypes and perform gene-drops separately for each user-identified population to avoid admixture problems. These meta statistics are available for chi-square association statistics and odds ratios and the MC procedure is used to access significance [40].

### Monte Carlo procedure

For each individual with sufficient genotype data (user-defined percent threshold), full-length MLE haplotype pairs are estimated as described above. Individuals with less than the specified percent threshold of genotype data are coded as completely missing. Association statistics of interest are calculated on the single SNPs, sub-haplotypes or full-length MLE haplotypes data as drawn from these full-length haplotypes. These statistics are called the observed statistics. For individuals included in the analyses but for whom missing data positions exist, these positions are imputed when the MLE haplotype pairs are estimated and therefore imputed data for these positions are used in full-haplotype, sub-haplotype and single SNP analyses.

The MC procedure generates a null distribution for each statistic to empirically determine significance. The MC procedure begins by creating "null multi-locus genotypic configurations" where the genetic data are simulated consistent with Mendelian inheritance but independent of the disease status. This is performed as follows. Haplotype-pairs are assigned to founders and independent individuals based on the estimated full length haplotype frequencies from the haplotype phasing step in observed data. Full length haplotypes are assigned to pedigree descendants using gene-dropping techniques based on Mendelian inheritance [46]. Hence, these null haplotype configurations are based on the same LD structures as the observed data. However, this creates full phase-known data. To properly match the observed data situation, the missing data structure of the observed data is imposed on each simulation and the remaining genetic data is considered as phase-unknown, thus creating a null multi-locus genotype configuration. Based on this null genotype configuration, MLE haplotype pairs are estimated. The phenotype data is the same as for the observed data. As was performed for the observed data, association statistics of interest are calculated for these null data and null statistics calculated. These null statistics are used to form a null distribution from which to assess the significance of the observed statistic.

It should be noted that although the Genie framework allows for the specification of non-zero recombination fractions (θ) between markers that our method used for haplotype frequencies and MLEs assumes no recombination between markers. Hence, the method is only relevant for limited genomic regions, such as small follow-up regions for GWAS (<1 Mb), candidate regions or sliding windows.

### Generation of simulated data for validation testing

We simulated data sets to assess the improvements of the phasing algorithm and to illustrate the validity and potential power of haplotype association testing using hapMC, particularly for analyzing mixed structured resources. To simulate SNP data under realistic conditions, HapMap CEPH Utah data was used for allele frequencies and LD structure. We chose an 18 kb region on chromosome 2 (230,976,558-30,994,737 bp) that contained 15 tightly linked (θ = 0) SNP markers with low pairwise r^2 ^values (similar to that expected from a regional tagging-SNP approach).

**Figure 2:**
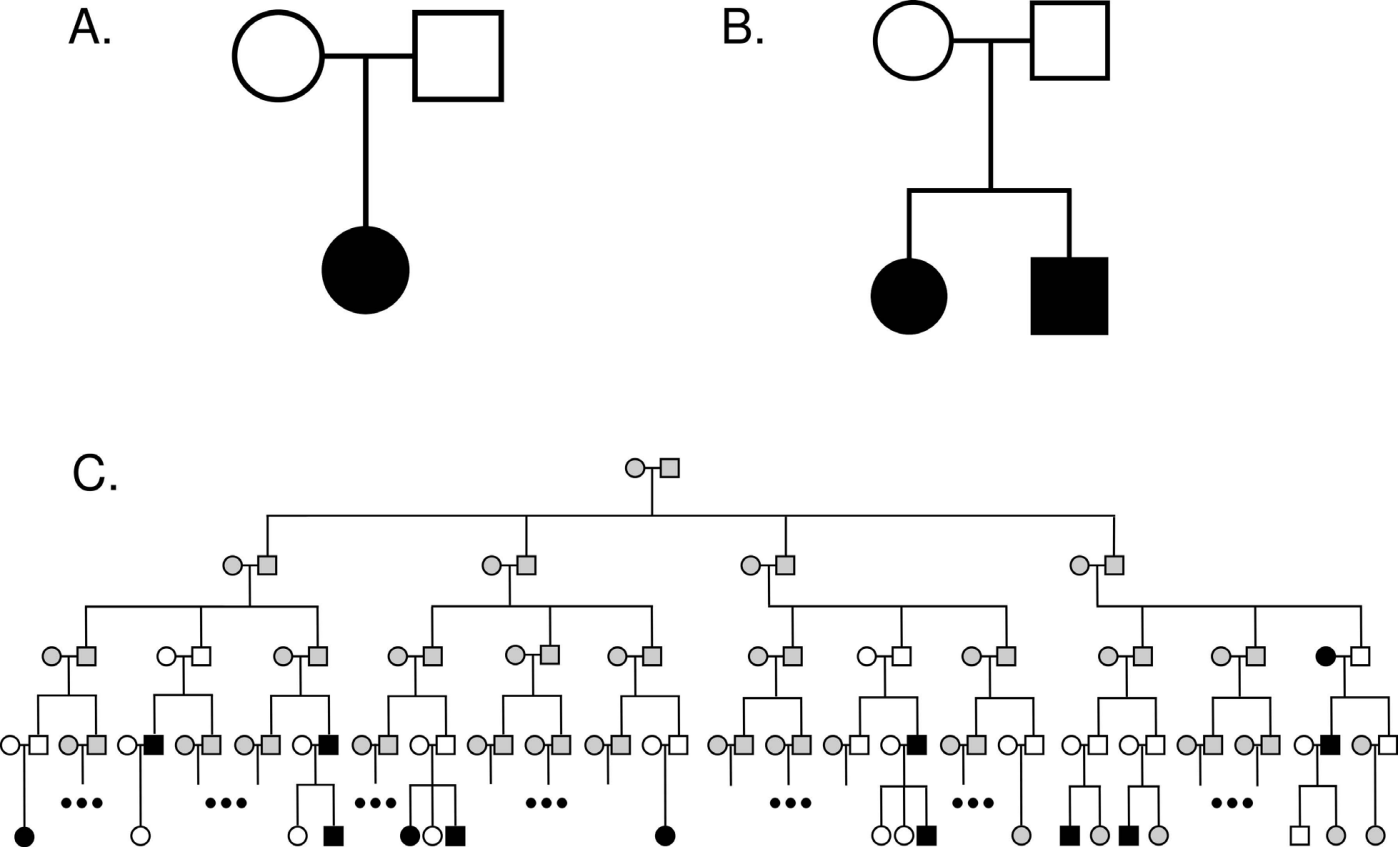
**Simulated pedigree structures**. A. Case-offspring trios (TRIO) B. Affected sib-pairs with parents (ASP) C. Five generation large pedigrees (LP). Black filled shapes are affected individuals (cases), white filled shapes are unaffected (controls) and grey filled are unknown.

Our family-based data sets were: (1) TRIOS -500 case-parent trios including a total of 500 cases and 1,000 parental controls; (2) ASPs -250 affected sib-pairs with parents including a total of 500 cases and 500 parental controls; (3) LP -large pedigrees (see Figure 2). The large, extended five-generation pedigrees were simulated to be high-risk (at least 14 cases were required). This structure was selected to mimic the data that would be available for large "linkage-like" pedigrees. All individuals in the top two generations were considered missing. All affected individuals (cases) were considered sampled. Regarding unaffected relatives (family controls), two LP data sets were generated: LP1 and LP2 to match the total numbers of cases and controls in the TRIO and ASP data sets. Hence, for LP1 sufficient LPs were generated to result in ~500 cases and ~1,000 controls and for LP2 the totals were ~500 cases and ~500 controls. In both LP1 and LP2, 80% of the family controls were close relatives to an affected individual (50% parents, 30% siblings) and the remaining 20% were beyond first degree relatives. We also simulated a fourth data set: (4) an independent case-control (CC) data set comprised of 500 cases and 500 controls.

Founder individuals were assigned haplotypes based on the genetic characteristics (allele frequencies and LD structure) of the selected chromosome 2 region. Next, a haplotype was selected to be the "risk haplotype" (we considered haplotype frequencies ranging between 0.04-0.17), which we assumed to have a haplotype r^2 ^of 0.8 with the underlying disease SNP (dSNP) allele. Conditional on each haplotype in the founders and an r^2 ^= 0.8, a dSNP allele was assigned to each founder haplotype. Descendants were then assigned haplotypes (including dSNP genotype) based on Mendelian inheritance rules using gene-dropping techniques. The dSNP genotypes were then removed. We assigned phenotypes under the null hypothesis of no association and under various alternate genetic models (see below). For the null scenario, affection status was randomly assigned to individuals independent of genetic data and based on a 5% sporadic rate for case-control and nuclear family data [47]. For null large pedigree simulations, phenotype clustering was simulated based on an alternate model, but genotypes were assigned independent of this phenotype. All alternative genetic models considered included a 5% sporadic rate and multiplicative genotypic relative risks at the dSNP ranging from 1.2 to 2.0. Simulations were repeated until a sufficient number of families of the types required were generated to form the data set. Each data set was replicated 500 times for the investigations of power and validity.

### Phasing comparison

To explore the properties of the new phasing algorithm, we considered four data sets all simulated under the null: 500 independent cases and 500 independent controls (CC); 500 TRIOs (500 cases and 1000 parental controls); 250 ASPs (500 cases and 500 parental controls); and the LP1 data set with ~500 cases and ~1000 controls. We considered marker sets comprised of 5, 10 and 15 SNP loci, and for those individuals with genotype data, we considered missing SNP rates of 0%, 5%, 10%, and 15%. For each of the four data sets, we examined the time to phase the observed data and the accuracy of the MLE haplotypes compared to the known true haplotypes using our new pedigree-informed phasing algorithm and a population-based EM phasing method that ignores relationships (GCHap) [48]. For independent individuals and nuclear family structures we also performed HAPLORE [19] for comparison. The same parameters for partition length (5 loci), overlap between partitions (1 locus), haplotype frequency cutoff (1 × 10^-6^) and haplotype buffer size (25 haplotypes beyond the cutoff) were used in our algorithm and HAPLORE. This process was repeated for five replicates to gain increased accuracy.

It is important to note that in our MC procedure we perform MLE haplotype estimation for both the real data and for each set of null data. Hence, it is imperative that the phasing step is efficient to gain reasonable run times.

### Power and validity

For each simulated data set (CC, TRIO, ASP, LP) we investigated the validity of haplotype analyses using hapMC based on pedigree-naïve and pedigree-informed (new algorithm) MLEs for three statistics: Cochran-Armitage test for trend using explicit control, Cochran-Armitage test for trend using pseudocontrols and also the TDT statistic[49,50], where applicable. Power was also assessed for the new pedigree-informed algorithm for a variety of genetic models. In addition, we illustrate the power and validity of haplotype association analysis for mixed resources of different structures consisting of each of the family data sets combined with the independent cases and controls: TRIOCC, ASPCC, LP1CC, and LP2CC.

Power and validity were estimated using 1,000 replicates. For each replicate, the specific "risk haplotype" was tested and assessed for significance using 1,000 null configurations in the MC procedure.

## Abbreviations

GWAS: genomewide-association studies; EM: Expectation-Maximization; MCMC: Markov chain Monte Carlo; LD: linkage disequilibrium; SNP: single nucleotide polymorphism; MC: Monte Carlo; TDT: transmission disequilibrium test; MLE: maximum likelihood estimate; HWE: Hardy Weinberg equilibrium; PL-EM: partition ligation expectation-maximization; CEPH: Centre d'Etude du Polymorphisme Humain; dSNP: disease single nucleotide polymorphism; ASP: affected sib-pair; LP: large pedigree; CC: case-control; EC: explicit control; PC: pseudo control

## Authors' contributions

RA designed and programmed the software, drafted the manuscript and performed the statistical analyses. JW contributed to the programming of hapMC and participated in the design of the software. AT contributed to the methodological development. NJC conceived of the methodology and helped to draft the manuscript. All authors read and approved the final manuscript.
